# Evaluation of the performance of biological drugs in the treatment of ankylosing spondylitis: cohort study, Minas Gerais, 2018-2023

**DOI:** 10.1590/S2237-96222025v34e20240116.en

**Published:** 2025-09-08

**Authors:** Bárbara Rodrigues Alvernaz dos Santos, Gerusa Araújo de Oliveira, Grazielle Dias Silva, Jessica Barreto Ribeiro dos Santos, Marina Morgado Garcia, Augusto Afonso Guerra Junior, Francisco de Assis Acurcio, Juliana Alvares-Teodoro

**Affiliations:** 1 Universidade Federal de Minas Gerais, Departamento de Farmácia Social, Belo Horizonte, MG, Brazil; 2 Universidade Federal do Espírito Santo, Alegre, ES, Brazil; 3 Secretaria de Estado de Saúde de Minas Gerais, Belo Horizonte, MG, Brazil

**Keywords:** Ankylosing Spondylitis, Effectiveness, Biological Products, Unified Health System, Cohort Studies, Espondilitis anquilosante, Efectividad, Productos Biológicos, Sistema Único de Salud, Estudios de cohorte

## Abstract

**Objectives:**

To assess the persistence and time until discontinuation of biological drugs used by people with ankylosing spondylitis treated in the Brazilian Unified Health System and to investigate the factors associated with them.

**Methods:**

Data were collected from an open historical cohort between 2018 and 2023 on the administrative processes required for requesting medicines from the specialized component of pharmaceutical assistance in Minas Gerais, Brazil. Sociodemographic and clinical data and treatment history were collected. A descriptive analysis of the variables was performed. The Cox proportional hazards model was used to identify factors associated with time to discontinuation of the first biological drug. The logistic regression model was used to evaluate the factors associated with persistence at 12 and 24 months of treatment.

**Results:**

A total of 530 participants were included, with an average age of 46 years, of which 50.0% were male. Adalimumab and golimumab were the most prescribed drugs. The mean follow-up time was 26.4 months, and the median time to discontinuation was 34 months. Persistence at 12 and 24 months was 82.0% and 63.0%. No significant differences were observed between biological drugs.

**Conclusion:**

The analysis of persistence and time to discontinuation showed no differences between the drugs, which justified their maintenance in the first line of treatment of the protocol. Factors associated with the clinical profile of users may be related to differences in effectiveness, but more in-depth studies are needed to confirm these results, given the limitations of using administrative data in epidemiological research.

## Resumen

**Objetivos:**

Evaluar la persistencia y el tiempo hasta la suspensión de los medicamentos biológicos utilizados por personas con espondilitis anquilosante atendidas en el Sistema Único de Salud e investigar los factores asociados a ellos.

**Métodos:**

A través de una cohorte histórica abierta, entre 2018 y 2023, se recolectaron datos sobre los procesos administrativos de solicitud de medicamentos del componente especializado de la asistencia farmacéutica en Minas Gerais. Se recogieron datos sociodemográficos, clínicos e historial de tratamiento. Se realizó un análisis descriptivo de las variables. Se utilizó el modelo de riesgos proporcionales de Cox para identificar los factores asociados con el tiempo hasta la interrupción del primer fármaco biológico. Se utilizó el modelo de regresión logística para evaluar los factores asociados a la persistencia a los 12 y 24 meses de tratamiento.

**Resultados:**

Se incluyeron 530 participantes, con una edad media de 46 años, de los cuales el 50,0% eran varones. Adalimumab y golimumab fueron los medicamentos más prescritos. El tiempo medio de seguimiento fue de 26,4 meses y el tiempo medio hasta la interrupción fue de 34 meses. La persistencia a los 12 y 24 meses fue del 82,0% y 63,0%. No se observaron diferencias significativas entre los fármacos biológicos.

**Conclusión:**

El análisis de persistencia y tiempo hasta la suspensión no mostró diferencias entre los fármacos, lo que justificó su mantenimiento en la primera línea de tratamiento del protocolo. Los factores asociados al perfil clínico de los usuarios pueden estar relacionados con diferencias en la efectividad, pero se necesitan estudios más profundos para confirmar estos resultados dadas las limitaciones del uso de datos administrativos en la investigación epidemiológica.

**Ethical aspects**

This research respected ethical principles, having obtained the following approval data:

Research Ethics Committee: Universidade Federal de Minas Gerais

Opinion number: 4.382.036

Approval date: 5/11/2020

Certificate of Submission for Ethical Appraisal: 36874120.5.0000.5149

Informed Consent Form: Exempted.

## Introduction

Spondyloarthritis is a heterogeneous group of rheumatic diseases with chronic inflammatory features that share clinical features, including asymmetric peripheral arthritis, enthesitis, dactylitis, and tenosynovitis (1,2). Ankylosing spondylitis is the main subtype of spondyloarthritis and mainly affects young people and adults, with a peak incidence between the ages of 20 and 40. Clinical manifestations include axial symptoms, such as inflammatory low back pain, and peripheral symptoms, such as arthritis, enthesitis, and dactylitis. (1,2).

The diagnosis of the disease can be performed using the Assessment of SpondyloArthritis International Society and the modified New York criteria. To measure disease activity and achieve therapeutic goals, including reducing disease activity, the Bath Ankylosing Spondylitis Activity Index and Ankylosing Spondylitis Disease Activity Score are the most commonly used tools (3,4).

In the Brazilian Unified Health System (SUS), the treatment of ankylosing spondylitis is performed based on the guidelines of the Clinical Protocol and Therapeutic Guidelines (5). The first stages of treatment include the use of nonsteroidal anti-inflammatory drugs, glucocorticoids and synthetic disease-modifying antirheumatic drugs, sulfasalazine and methotrexate (5). In the event of failure or contraindication to methotrexate or sulfasalazine, biological disease-modifying anti-inflammatory drugs may be indicated (5). In the SUS (Brazilian Unified Health System), biological drugs are part of the range of medicines in the specialized component of pharmaceutical assistance. Biological drugs include tumor necrosis factor inhibitors (adalimumab, etanercept, infliximab, golimumab, certolizumab pegol) and interleukin 17-A inhibitor (secukinumab). 

Despite the numerous therapeutic possibilities, a major challenge is maintaining treatment for a prolonged period. Persistence, defined as the proportion of individuals who remain on treatment over a defined period of time, is a combined measure of effectiveness, safety and adherence to treatment that can be used to assess the performance of medications in a real-world setting (6-9). 

In the SUS, biological drugs for the treatment of spondylitis were incorporated based on the results of pivotal clinical trials which, by their very nature, mean that decisions are permeated by uncertainties regarding the effectiveness and safety of these drugs. Monitoring their performance after incorporation is crucial to ensuring access to effective and safe medicines, in addition to maintaining the sustainability of the SUS, given its high cost (10,11). The information obtained through the administrative processes of the specialized component of pharmaceutical assistance are valuable tools for monitoring and evaluating the performance of technologies incorporated into the SUS. 

This study aimed to evaluate the persistence and time to discontinuation of biological drugs used by people with ankylosing spondylitis treated at SUS and to investigate the factors associated with them. We sought to answer the following questions: “*What is the persistence at 12 and 24 months, and what is the time to discontinuation of biological drugs used in the treatment of ankylosing spondylitis in the SUS? What are the factors associated with persistence and time to discontinuation*?”

## Methods

### Study design

This was an open historical cohort, constructed with secondary data. These came from the administrative processes for requesting medicines from the specialized component of pharmaceutical assistance in Minas Gerais. Users were followed from June 2018 to September 2023. Data collection took place between 2020 and 2023. 

The study included all adults (>18 years old) resident in Minas Gerais, diagnosed with ankylosing spondylitis by the International Statistical Classification of Diseases and Related Health Problems (ICD-10) M45, whose requests for biological drugs (adalimumab, etanercept, infliximab, golimumab, certolizumab pegol or secukinumab) were granted by the aforementioned component. Users of any biological drug previously mentioned through private funding or donation were excluded. Individuals who used biological drugs before entering this cohort, prior to June 2018, and people who were using biological drugs to treat other rheumatic conditions were also excluded.

## Data collection

For data collection, a standardized form was developed, created from the information present in the medication request, evaluation, and authorization report for requesting medications from the specialized component of pharmaceutical assistance from the Minas Gerais State Health Department. The form was submitted for validation by experts, who assessed its clarity, relevance, and comprehensiveness in relation to the information required for the study. This step was performed with the aim of ensuring that the instrument was suitable for capturing essential data in a structured and consistent manner, minimizing biases associated with the interpretation of data by researchers or the omission of vital information.

Sociodemographic (age, sex, municipality of residence and race/skin color) and clinical data (duration of disease, modified New York criteria, Assessment of SpondyloArthritis International Society, medical report on clinical symptoms and history of treatment for ankylosing spondylitis) were collected. Socioeconomic status was assessed using the Gini index. In this study, the Gini index was analyzed for each municipality of residence of the individuals included in this cohort (217 municipalities).

Through the integrated pharmaceutical assistance management system, drug dispensing data were obtained. The dispensation was updated until September 2023, which allowed each participant to be monitored for at least 12 months from the date of the first biological drug dispensation. 

Measures were taken to avoid the risk of bias related to information by collecting data from standardized and verified sources. As a strategy to ensure the accuracy of the information, data were checked in duplicate for the exposure and outcome variables.

### Outcomes analyzed

The time to discontinuation was considered as the main outcome and defined as the time until dispensing was interrupted, considering the period of 60 days without the supply of the drug after the presumed end of the prescription, that is, the last day with presumed availability of the drug (grace period) (5,9,12). Users were classified as having discontinued treatment when they stopped taking or switched to another biological drug. 

Persistence – being on treatment – was assessed at 12 and 24 months after the first dispensing. In the analysis of persistence over 12 months, users who had the opportunity to be monitored for this period were included. This was also done for the calculation of persistence at 24 months. This measure allowed us to analyze the proportion of participants who persisted in treatment, after one and two years, and the associated factors were verified. 

### Statistical analysis

Descriptive statistics were applied to describe the demographic and clinical characteristics of the participants. Proportions were used for categorical variables such as sex, race/skin color, Gini index, modified New York criteria, Assessment of Spondylarthritis International Society, and presence of HLA-B27. For continuous variables, such as age, duration of illness and duration of use of the biological drug, dispersion measures were used.

The occurrence of missing data in some variables was less than 5.0%, therefore there was no need for data imputation, given the reduced impact on statistical analyses. To compare the proportions of switching or discontinuation between medications, Pearson’s chi-square test was used. 

The Cox proportional hazards model was used to identify factors associated with time to discontinuation of the first biological drug. The model considered the adjusted hazard ratio, along with the 95% confidence interval. The variables included in the Cox model were age, sex, race, socioeconomic indicator, HLA-B27 status, disease duration, clinical symptoms, and medication use. The Kaplan-Meier survival curve was generated to compare the time to discontinuation of treatment, considering the presence of HLA-B27. Differences between groups were analyzed using the log-rank test throughout the period evaluated. 

To assess the existence of an association between demographic and clinical variables and persistence in treatment, univariate analyses were conducted using Pearson’s chi-square test. Multivariate analysis was performed using the logistic regression model. To assemble the model, the variables that presented p-value<0.200 in the univariate model were used. The variable selection method was followed until the final model was obtained. Using the Hosmer-Lemeshow test, the adequacy of the model was assessed. 

A significance level (p-value 0.050) was used for multivariate analysis and Pearson’s chi-square test. All analyses were performed using the IBM SPSS Statistics, version 26.

## Results

Between 2018 and 2023, 530 participants were monitored and analyzed. The mean follow-up time was 26 months. 

Among the participants monitored, 265 (50.0%) were male and 169 (31.9%) declared themselves to be white (Table 1). The mean age of participants was 46.4 years (standard deviation ±13.17). The mean time of disease until the prescription of the first biological drug was 5.9 years (standard deviation ±7.90). The mean Bath Ankylosing Spondylitis Activity Index of participants in this cohort was 6.9 points (standard deviation±1.52), which indicated active disease. High disease activity was also identified using the Ankylosing Spondylitis Disease Activity Score tool, with mean values of 4.7 (standard deviation ±2.1) (Table 1). 

**Table 1:** Sociodemographic and clinical characteristics and medication use profile of participants with ankylosing spondylitis using biological drugs. Minas Gerais, 2018-2023 (n=530)

Characteristics	n (%)
Gender	
Male	265 (50.0)
Female	265 (50.0)
**Race/skin color**	
White	169 (31.9)
Brown/black	113 (21.3)
Other/ignored information	248 (46.8)
**Age group** (years)	
18-44	235 (44.3)
45-59	206 (38.9)
≥60	89 (16.8)
**Gini Index**	
0.0000-0.4999	298 (56.2)
0.5000-1.000	232 (43.8)
**Length of illness** (years) 0-5 ≥6	230 (43.4) 300 (56.6)
**Clinical condition**	
Enthesitis	430 (81.3)
Peripheral arthritis	114 (21.5)
Inflammatory low back pain with onset before age 45^a^	503 (94.9)
Sacroiliitis in imaging tests^a^	478 (90.2)
Presence of HLA-B27^a^	300 (56.6)
Low back pain for more than three months^b^	315 (59.4)
Limitation of lumbar spine movements^b^	353 (66.6)
Decreased chest expansion^b^	166 (31.3)
X-ray showing bilateral sacroiliitis of grades 3-4^b^	366 (69.1)
X-ray showing unilateral sacroiliitis of grades 3 or 4^b^	104 (19.6)
**Previous use of drugs for ankylosing spondylitis**	
Non-steroidal anti-inflammatory drugs	525 (99.1)
Corticosteroids	137 (25.8)
Methotrexate	210 (39.6)
Sulfasalazine	202 (38.1)
Leflunomide	18 (3.4)
Hydroxychloroquine	13 (2.5)
**First biological drug**	
Adalimumab	198 (37.4)
Etanercept	30 (5.7)
Infliximab	80 (15.1)
Certolizumab pegol	53 (10.0)
Golimumab	159 (30.0)
Secukinumab	10 (1.9)

^a^Assessment of SpondyloArthritis International Society; ^b^Modified New York criteria.

The drugs adalimumab and golimumab were the most prescribed, corresponding to 37.4% and 30.0%. Etanercept was the least prescribed tumor necrosis factor inhibitor, 5.7% (Table 1).

Participants using infliximab had a higher proportion of discontinuation (27.5%). Pearson’s chi-square test demonstrated statistically significant differences for treatment discontinuation for the drugs infliximab (higher discontinuation rate) and golimumab (lower discontinuation rate) (p-value<0.050). For the drug switching outcome, no statistically significant differences were found (Table 2).

**Table 2:** Number and proportion of drug switches and discontinuation of the first prescribed biological drug. Minas Gerais, 2018-2023 (n=530)

Drug	Prescribed	Switch	Interruption
	n	n (%)	n (%)
Adalimumab	198	43 (21.7)	34 (17.2)
Etanercept	30	9 (30.0)	5 (16.7)
Infliximab	80	10 (12.5)	22 (27.5)^a^
Certolizumab pegol	53	12 (22.6)	8 (15.1)
Golimumab	159	34 (21.4)	19 (11.9)^a^
Secukinumab	10	1 (10.0)	0 (0.0)
Total	530	109 (20.6)	88 (16.6)

^a^p-value<0.050 in Pearson’s chi-square test.

**Figure 1:**
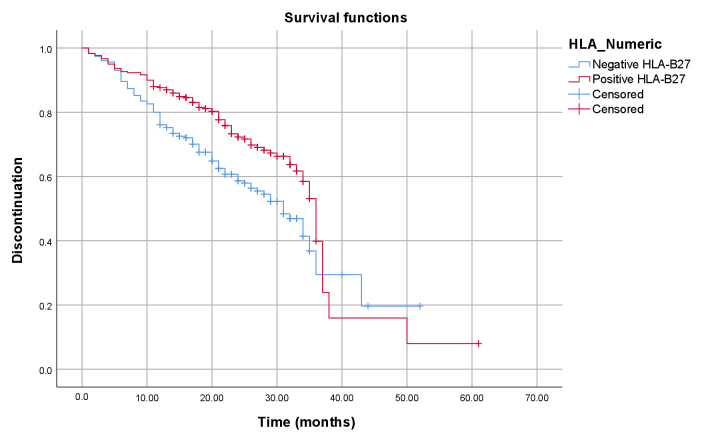
Kaplan-Meier with estimated therapeutic discontinuation associated with the presence of HLA-B27 in 60 months. Minas Gerais, 2018-2023 (n=530)

The median time to discontinuation of treatment was 34.0 months. In the Cox proportional hazards model, the risk of discontinuation of therapy was analyzed as a time-dependent variable. The analysis revealed that race/skin color, the presence of HLA-B27 and inflammatory low back pain for more than three months presented a greater risk of treatment discontinuation. Race/skin color showed a statistically significant difference (p-value 0.045) with adjusted hazard ratio indicating that individuals with Brown or Black race/skin color had a 35.0% lower risk of discontinuing treatment compared to those of other skin colors. The presence of HLA-B27 also demonstrated a significant association (p-value 0.001), with a 73.0% higher risk of treatment discontinuation among individuals with positive HLA-B27 (Figure 1). Inflammatory low back pain for more than three months, although with a tendency towards marginal significance (p-value 0.053), increased the risk of treatment discontinuation by 40.0% (Table 3).

**Table 3:** Hazard ratio (HR) and 95% confidence interval (95%CI) adjusted for variables associated with discontinuation of therapy: Cox regression model. Minas Gerais, 2018-2023 (n=197)

Variables	Discontinuation rate n (%)	Median time to discontinuation (months)	p-value	Adjusted HR (95%CI)^a^
Gender			0.237	
Female	104 (39.2)	33.0 (31.15; 34.85)		
Male	93 (35.1)	34.0 (31.18; 36.82)		
**Age group** (years)			0.672	
18-44	85 (36.2)	33. 0 (30.87; 35.13)		
45-59	80 (38.8)	35.0 (30.90; 39.10)		
>60	32 (35.9)	33.0 (30.10; 5.90)		
**Race/skin color**			0.045	
White	73 (43.2)	31.0 (25.15; 36.85)		1
Brown/black	37 (32.7)	34.0 (29.61; 38.39)		0.65 (0.43; 0.96)
Other/unknown	87 (35.1)	33.0 (30.93; 35.07)		0.76 (0.55; 1.04)
**Gini index**			0.346	
0.0000-0.4999	108 (36.2)	35.0 (32.7; 37.33)		
0.5000-1.000	89 (38.4)	33.0 (29.95; 36.05)		
**HLA-B27**			<0.001	
No	104 (45.2)	30.0 (25.92; 34.09)		1
Yes	93 (31.0)	35.0 (32.98; 37.02)		1.73 (1.30; 2.30)
**Length of illness** (years)			0.896	
0-5	88 (38.3)	34.0 (30.7; 37.3)		
≥6	109 (36.3)	33.0 (30.7; 35.3)		
**Inflammatory low back pain for more than three months**			0.053	
No	88 (40.9)	33.0 (27.6; 38.4)		1
Yes	109 (34.6)	34.0 (32.2; 35.8)		1.40 (1.06; 1.86)
**Limitations of lumbar spine movement**			0.208	
Yes	126 (35.7)	33.0 (31.6; 34.4)		
No	71 (40.1)	34.0 (24.2; 43.8)		
**Decreased chest expansion**			0.062	
No	143 (39.3)	34.0 (31.5; 36.5)		1
Yes	54 (32.5)	33.0 (30.7; 35.3)		1.40 (1.03; 1.95)
**Unilateral sacroiliitis**			0.610	
Yes	35 (33.7)	32.0 (29.3; 34.7)		
No	162 (38.0)	35.0 (32.01; 37.9)		
**Bilateral sacroiliitis**			0.273	
Yes	129 (35.2)	34.0 (32.4; 35.5)		
No	68 (41.5)	33.0 (26.1; 39.8)		
**Previous use of corticosteroids**			0.121	
Yes	48 (35.0)	34.0 (32.5; 35.4)		
No	149 (37.9)	33.0 (30.1; 35.9)		
**First biological drug**			0.704	
Adalimumab	77 (38.9)	33.0 (28.1; 37.9)		
Etanercept	14 (46.7)	29.0 (17.5; 40.5)		
Infliximab	32 (40.0)	35.0 (26.2; 43.8)		
Certolizumab	20 (37.7)	33.0 (25.4; 40.6)		
Golimumab	53 (33.3)	34.0 (32.3; 35.7)		
Secukinumab	1 (10.0)	-		

^a^Multivariate analysis.

When analyzing the relationship of baseline demographic and clinical variables with persistence at 12 and 24 months of follow-up, statistically significant differences were observed in participants with HLA-B27, inflammatory low back pain for more than three months and decreased chest expansion (p-value≤0.050) (Table 4). The results in 12 months revealed that patients with decreased chest expansion had a 55.0% reduction in the chances of therapeutic persistence. HLA-B27 was identified as a risk factor, showing a 60.0% reduction in the chances of therapeutic persistence compared to those with a negative test. The presence of inflammatory low back pain for more than three months was associated with a 48.0% decrease in the chances of therapeutic persistence. In 24 months of follow-up, only the clinical symptom of chest expansion demonstrated a significant association with persistence to treatment (p-value 0.052) (Table 4).

**Table 4:** Odds ratio (OR) and 95% confidence interval (95%CI) adjusted for variables associated with persistence at 12 and 24 months. Minas Gerais, 2018-2023 (n=530)

Variables	Persistence in 12 months (n=530) %	p-value	Adjusted OR (95%CI)^a^	Persistence in 24 months (n=332) %	p-value	Adjusted OR (95%CI)^a^
General	82.1			63.0		
Gender		0.910			0.610	
Female	81.9			61.6		
Male	82.3			64.3		
**Age group** (years)		0.418			0.997	
18-44	83.8			63.0		
45-59	82.0			63.1		
>60	77.5			62.5		
**Race/skin color**		0.664			0.393	
White	81.1			58.3		
Brown/black	85.0			62.3		
Other/unknown	81.5			66.7		
**Gini index**		0.747			0.237	
0.0000-0.4999	82.6			65.5		
0.5000-1.000	81.5			59.1		
HLA-B27		0.001			0.066	
Yes	87.0		1	67.2		1
No	75.7		0.40 (0.25; 0.64)	57.3		0.63 (0.40; 0.99)
**Length of illness** (years)		0.334			0.330	
0-5	80.7			60.7		
≥6	83.9			66.0		
**Inflammatory low back pain for more than three months**		0.016			0.141	
Yes	85.4		1	66.2		
No	77.2		0.52 (0.33; 0.82)	58.2		
**Limitations of lumbar spine movement**		0.132			0.414	
Yes	83.9			64.4		
No	78.5			59.8		
**Decreased chest expansion**		0.017			0.052	
Yes	88.0		1	70.4		1
No	79.4		0.45 (0.26; 0.79)	59.4		0.59 (0.36; 0.96)
**Unilateral sacroiliitis**		0.108			0.510	
Yes	87.5			66.7		
No	80.8			62.1		
**Bilateral sacroiliitis**		0.377			0.168	
Yes	83.1			65.4		
No	79.9			57.4		
**Previous use of corticosteroids**		0.508			0.044	
Yes	83.9			70.8		
No	81.4			59.3		
**First biological drug**		0.412				
Adalimumab	82.3					
Etanercept	73.3					
Infliximab	85.0					
Certolizumab	77.4					
Golimumab	82.4					
Secukinumab	100.0					

## Discussion

The performance of biological drugs used in the treatment of ankylosing spondylitis in the SUS, in Minas Gerais, was evaluated based on data obtained from the administrative processes of the specialized component of pharmaceutical assistance. 

The participants who comprised this cohort had a mean age and gender distribution similar to those previously observed in Brazil (13,14). The average duration of the disease was close to that observed in other Brazilian cohorts, in which the average duration of the disease was 7.6 years (13,14). The results confirmed that the onset of spondylitis occurs, on average, in the third decade of life, with clinical worsening manifesting approximately six years after diagnosis.

Most participants declared themselves white, and some did not want to declare their skin color. Between 53.0% and 56.0% of users with ankylosing spondylitis do not identify themselves as white (14,15). Globally, available epidemiological data suggest a higher prevalence of the disease in white people. However, these studies were mostly carried out in European and North American countries, with populations that are not very comparable to the Brazilian population. (16,17). The data gap on race/skin color in the Brazilian context has limited the understanding of the impact of the disease on this population group.

The Gini index was used to represent the socioeconomic status of participants based on income inequality in their municipality of residence. Its interpretation can be added to the human development index, which measures municipal development through health, education, and per capita income indicators. In Minas Gerais, the human development index in 2021 was 0.774, which placed the state among the four with the highest index in the country (18). This result was in line with the average distribution achieved by the Gini index in this study.

Just over half of the participants had HLA-B27. This number diverged from the findings described in the international literature. A systematic review summarized the results from North American and European countries (19). In this study, 70.0% and 94.0% of participants had HLA-B27 (19). In Spain, it was also observed that 75.0% of participants in a given cohort had HLA-B27 (20). In this study, the low prevalence of HLA-B27 may be associated with specific genetic and ethnic factors of the population evaluated.

Other analyses involving the antigen were conducted. Among these, HLA-B27 stands out as a factor associated with longer time to discontinuation and greater persistence in treatment. The role of HLA-B27 in the pathogenesis of the disease is discussed, however the relationship between the presence of HLA-B27 and the worse prognosis of the disease is little addressed. The presence of the HLA-B27 allele demonstrated a worse prognosis and earlier onset of disease symptoms, as demonstrated in studies with samples from 150 and 1,235 participants (21,22).

Adalimumab was the most prescribed as first choice (13,14,23). Maintaining adalimumab as the first option may indicate the prescribers’ preference for this treatment and the benefits achieved with its use both nationally and internationally (23).

The cases of secukinumab prescription as the first choice are at odds with the criteria established in the Clinical Protocol and Therapeutic Guidelines (5). The justifications for using the medication included a positive tuberculin skin test result, extra-articular manifestations, failure to use non-steroidal anti-inflammatory drugs and synthetic disease-modifying drugs. The protocol recommends the use of secukinumab exclusively after therapeutic failure with a tumor necrosis factor inhibitor (5). The use of secukinumab in the first line of treatment and the benefits related to the use of the drug were reported, including greater persistence and improvement in disease activity (24). 

The 2016 Assessment of SpondyloArthritis International Society and European League Against Rheumatism international recommendations approved the use of secukinumab only for people with axial spondyloarthritis and radiographic sacroiliitis. In the 2022 update, the criteria were changed, and secukinumab was indicated as first-line treatment for axial spondyloarthritis (25). This update reinforced that the drug has been standing out in the treatment of the disease, proving to be as effective and safe as tumor necrosis factor inhibitors.

Medication switching occurred more frequently among participants using etanercept. In this study, the observed discontinuation rate was lower than previously reported (13.26). The main cause of discontinuation (50.0%) as observed in this study was related to therapeutic inefficiency, while 30.0% were attributed to adverse drug reactions (26). 

In this cohort, people with HLA-B27 or inflammatory low back pain for more than three months were at higher risk for treatment discontinuation. The results suggested an association between disease severity and worse performance of some drugs, highlighting the importance of considering clinical factors in managing treatment persistence. Discontinuation or interruption of therapy may result in increased public spending, given the possible worsening of these users’ health. Strategies such as educating users about the risks of discontinuing therapy may be useful. For more serious cases, in which the disease has a worse prognosis, it is essential to reinforce the need for persistence with treatment, aiming to stabilize the condition and avoid long-term complications. 

Treatment persistence can be considered an indicator of effectiveness, tolerance and adherence to drug therapy (8.27). In a cohort of 19,319 Brazilian and Canadian users, it was found that the average persistence time in using adalimumab, etanercept and infliximab by participants was ten months (14). It was shown that 52.0% to 65.0% of participants using infliximab, etanercept and adalimumab persisted until the first year (14). In another Brazilian cohort, with 1,251 users, 80.0% of individuals persisted in the first year of follow-up, while 60.0% were using the biological drug at the end of the second year (13). Despite the differences found between the studies, the lack of persistence after the first year of treatment raises questions about the possible lack of therapeutic effectiveness of these medications after one year of use.

This study had limitations inherent to real-world research, especially those that use secondary data. The data analyzed were obtained from administrative processes for requesting high-cost medications. Specialist doctors complete these documents, and their information is certified by the results of imaging and laboratory tests attached to the process. According to the protocol requirements, the history of previous use of biological drugs must be recorded in the request report. Possible inconsistencies or omissions in filling out the form may limit the completeness and accuracy of the data. 

Measures to reduce the risk of information bias were applied, but it is important to emphasize that the absence of clinical data, such as comorbidities and use of other medications, limits the detailed analysis of clinical factors, which can directly impact treatment persistence. In addition to the burden of comorbidities, behavioral factors can also influence treatment persistence. Aspects such as adherence to treatment, regular access to health services and lifestyle habits can impact therapeutic outcomes but are not systematically documented in the administrative records used. These limitations must be considered when interpreting the findings.

Another limitation is associated with the way in which socioeconomic status was assessed, using exclusively the Gini index. Although this indicator measures income inequality within a city, it does not directly reflect absolute income, access to health services or the quality of life of individual users. The impossibility of collecting other indicators, such as per capita income, access to health services or educational level, is due to the limitation of the data available in the forms analyzed. The Gini index does not allow us to infer, for example, whether users with greater purchasing power and better access to healthcare are the main users of these therapies.

Minas Gerais, due to its territorial extension and diverse population, can reflect the national reality. Collected routinely, SUS administrative data are a valuable source for real-world studies, allowing a detailed view of the epidemiology and clinical status of users. Its analysis provides essential information about the effectiveness and safety of treatments, which helps in public health decision-making. The wide availability of these data enables more representative population studies, enabling the identification of health outcomes and risk factors with greater precision.

This study highlighted the usefulness of administrative data on pharmaceutical assistance as a tool for monitoring the clinical profile and therapeutic persistence of users with ankylosing spondylitis in the SUS. Clinical factors, such as HLA-B27 and disease symptoms, had a higher risk of patients discontinuing treatment with biological drugs. However, in 24-months follow-up, an association of persistence associated with positive HLA-B27 was observed. This may indicate that persistence with treatment may be time-dependent. On the other hand, demographic aspects, such as race/skin color, demonstrated protective effects. The observed patterns of prescription and switching of biological drugs pointed to variations in persistence and discontinuation rates, which may reflect differences in both clinical management and user characteristics. These findings reinforce the need to integrate clinical and sociodemographic factors in the personalized management of ankylosing spondylitis, contributing to better therapeutic outcomes. 

## Data Availability

The database and analysis codes used in the research are available upon request to the authors.
