# Supplementary material for: Evaluation of the performance of biological drugs in the treatment of ankylosing spondylitis: cohort study, Minas Gerais, 2018-2023
Source: Epidemiol Serv Saude. 2025 Sep 8;34:e20240116. doi: 10.1590/S2237-96222025v34e20240116.en (PMC12435891; doi:10.1590/S2237-96222025v34e20240116.en)
Supplement: Translation

# Translation: Avaliação do desempenho de medicamentos biológicos no tratamento da espondilite anquilosante: estudo de coorte, Minas Gerais, 2018-2023

**DOI:** 10.1590/S2237-96222025v34e20240116.pt

**Authors:** Bárbara Rodrigues Alvernaz dos Santos, Gerusa Araújo de Oliveira, Grazielle Dias Silva, Jessica Barreto Ribeiro dos Santos, Marina Morgado Garcia, Augusto Afonso Guerra Junior, Francisco de Assis Acurcio, Juliana Alvares-Teodoro

**Affiliations:** 1 Universidade Federal de Minas Gerais, Departamento de Farmácia Social, Belo Horizonte, MG, Brasil; 2 Universidade Federal do Espírito Santo, Alegre, ES, Brasil; 3 Secretaria de Estado de Saúde de Minas Gerais, Belo Horizonte, MG, Brasil

**Keywords:** Espondilite Anquilosante, Efetividade, Produtos Biológicos, Sistema Único de Saúde, Estudos de Coortes

## Abstract

**Objetivos:**

Avaliar a persistência e o tempo até a descontinuação de medicamentos biológicos utilizados por pessoas com espondilite anquilosante atendidas no Sistema Único de Saúde e investigar os fatores a eles associados.

**Métodos:**

Por meio de coorte histórica aberta, entre 2018 e 2023, foram coletados dados dos processos administrativos de solicitação de medicamentos do componente especializado da assistência farmacêutica de Minas Gerais. Coletaram-se os dados sociodemográficos e clínicos e o histórico de tratamento. Foi feita análise descritiva das variáveis. O modelo de riscos proporcionais de Cox foi utilizado para identificar fatores associados ao tempo até a descontinuação do primeiro medicamento biológico. Utilizou-se o modelo de regressão logística para avaliar os fatores associados à persistência em 12 e 24 meses de tratamento.

**Resultados:**

Incluíram-se 530 participantes, com idade média de 46 anos, dos quais 50,0% eram do sexo masculino. Adalimumabe e golimumabe foram os medicamentos mais prescritos. O tempo médio de acompanhamento foi 26,4 meses, e o tempo mediano até a descontinuação, 34 meses. A persistência em 12 e 24 meses foi 82,0% e 63,0%. Não foram observadas diferenças significativas entre os medicamentos biológicos.

**Conclusão:**

A análise de persistência e tempo até a descontinuação não mostrou diferenças entre os medicamentos, o que justificou sua manutenção na primeira linha de tratamento do protocolo. Fatores associados ao perfil clínico dos usuários podem estar relacionados a diferenças de efetividade, mas estudos mais aprofundados são necessários para confirmar esses resultados dadas as limitações do uso de dados administrativos em pesquisas epidemiológicas.

**Aspectos éticos**

A presente pesquisa respeitou os princípios éticos, obtendo os seguintes dados de aprovação:

Comitê de ética em pesquisa: Universidade Federal de Minas Gerais

Número do parecer: 4.382.036

Data de aprovação: 5/11/2020

Certificado de apresentação de apreciação ética: 36874120.5.0000.5149

Registro do consentimento livre e esclarecido: Dispensado.

## Introdução

As espondiloartrites são um grupo heterogêneo de doenças reumáticas de características inflamatórias crônicas que compartilham características clínicas, incluindo artrite periférica assimétrica, entesite, dactilite e tenossinovite (1,2). A espondilite anquilosante é o principal subtipo das espondiloartrites e acomete principalmente jovens e adultos, com pico de incidência dos 20 aos 40 anos. As manifestações clínicas incluem sintomas axiais, como dor lombar inflamatória e sintomas periféricos, a exemplo de artrite, entesite e dactilite (1,2).

O diagnóstico da doença pode ser realizado com o uso dos instrumentos Assessment of SpondyloArthritis International Society e os critérios modificados de Nova York. Para mensuração da atividade da doença e alcance dos objetivos terapêuticos, incluindo a redução da atividade da doença, as ferramentas Bath Ankylosing Spondylitis Activity Index e Ankylosing Spondylitis Disease Activity Score são as mais utilizadas (3,4).

No Sistema Único de Saúde (SUS), o tratamento da espondilite anquilosante é realizado a partir das orientações do Protocolo Clínico e Diretrizes Terapêuticas (5). As primeiras etapas do tratamento incluem o uso de anti-inflamatórios não esteroidais, glicocorticoides e modificadores do curso de doença sintéticos, sulfassalazina e metotrexato (5). Na ocorrência de falha ou contraindicação ao metotrexato ou à sulfassalazina, os medicamentos modificadores do curso de doença biológicos podem ser indicados (5). No SUS, os medicamentos biológicos fazem parte do elenco de medicamentos do componente especializado da assistência farmacêutica. Entre os medicamentos biológicos, estão os inibidores do fator de necrose tumoral (adalimumabe, etanercepte, infliximabe, golimumabe, certolizumabe pegol) e o inibidor da interleucina 17-A (secuquinumabe). 

Apesar das inúmeras possibilidades terapêuticas, um grande desafio é a manutenção do tratamento por período prolongado. A persistência, definida como a proporção de indivíduos que se mantêm em tratamento em determinado período definido de tempo, é uma medida combinada de efetividade, segurança e adesão ao tratamento que pode ser utilizada para avaliar o desempenho dos medicamentos em um cenário de mundo real (6-9). 

No SUS, os medicamentos biológicos para tratamento da espondilite foram incorporados com base nos resultados dos ensaios clínicos pivotais que, por sua própria natureza, fazem com que as decisões sejam permeadas por incertezas acerca da efetividade e segurança desses medicamentos. O monitoramento do seu desempenho pós-incorporação é crucial para a garantia de acesso a medicamentos efetivos e seguros, além da manutenção da sustentabilidade do SUS, tendo em vista o seu alto custo (10,11). As informações obtidas por meio dos processos administrativos do componente especializado da assistência farmacêutica são ferramentas valiosas para monitorar e avaliar o desempenho de tecnologias incorporadas ao SUS. 

Este estudo teve como objetivo avaliar a persistência e o tempo até a descontinuação de medicamentos biológicos utilizados por pessoas com espondilite anquilosante atendidas no SUS e investigar os fatores a eles associados. Buscou-se responder às seguintes perguntas: “Qual é a persistência, em 12 e 24 meses, e qual é o tempo até a descontinuação do uso dos medicamentos biológicos utilizados no tratamento da espondilite anquilosante no SUS? Quais são os fatores associados à persistência e ao tempo até a descontinuação?”.

## Métodos

### Desenho do estudo

Tratou-se de coorte histórica aberta, construída com dados secundários. Estes foram provenientes dos processos administrativos de solicitação de medicamentos do componente especializado da assistência farmacêutica de Minas Gerais. Os usuários foram acompanhados de junho de 2018 a setembro de 2023. A coleta de dados ocorreu entre 2020 e 2023. 

Foram incluídos todos os indivíduos adultos (>18 anos), residentes em Minas Gerais, diagnosticados com espondilite anquilosante pela Classificação Estatística Internacional de Doenças e Problemas Relacionados à Saúde (CID-10) M45, cujas solicitações de medicamentos biológicos (adalimumabe, etanercepte, infliximabe, golimumabe, certolizumabe pegol ou secuquinumabe) foram deferidos pelo referido componente. Foram excluídos usuários de qualquer medicamento biológico citado anteriormente por financiamento privado ou doação. Também não foram incluídos indivíduos que utilizaram medicamento biológico antes do período de entrada nesta coorte, anterior a junho de 2018, e pessoas que estivessem em uso de medicamentos biológicos para o tratamento de outras condições reumáticas.

### Coleta de dados

Para a coleta de dados, foi desenvolvido formulário próprio padronizado, criado a partir das informações presentes no laudo de solicitação, avaliação e autorização de medicamentos para solicitação de medicamentos do componente especializado da assistência farmacêutica da Secretaria de Estado de Saúde de Minas Gerais. O formulário foi submetido à validação por especialistas, que avaliaram sua clareza, relevância e abrangência em relação às informações necessárias para o estudo. Essa etapa foi realizada com o objetivo de garantir que o instrumento fosse adequado para captar os dados essenciais de forma estruturada e consistente, minimizando vieses associados à interpretação dos dados pelos pesquisadores ou à omissão de informações importantes.

Foram coletados dados sociodemográficos (idade, sexo, município de residência e raça/cor da pele) e clínicos (tempo de duração da doença, critérios modificados de Nova York, Assessment of SpondyloArthritis International Society, relato médico acerca dos sintomas clínicos e histórico de tratamento para espondilite anquilosante). O status socioeconômico foi avaliado por meio do índice de Gini. Neste estudo, foi analisado o índice de Gini para cada município de residência dos indivíduos incluídos nesta coorte (217 municípios).

Por meio do sistema integrado de gerenciamento da assistência farmacêutica, foram obtidos os dados de dispensação dos medicamentos. A dispensação foi atualizada até setembro de 2023, o que permitiu o acompanhamento de cada participante por, no mínimo, 12 meses a partir da data da dispensação do primeiro medicamento biológico. 

Foram adotadas medidas para evitar risco de viés relacionado à informação por meio da coleta de dados de fontes padronizadas e verificadas. Como estratégia para garantir a acurácia das informações, foi realizada a conferência dos dados em duplicata para as variáveis de exposição e desfecho.

### Desfechos analisados

O tempo até a descontinuação foi considerado como desfecho principal e definido como o tempo até a interrupção da dispensação, considerando o período de 60 dias sem o fornecimento do medicamento após o término presumido da prescrição, ou seja, o último dia com disponibilidade presumida de medicamentos (período de carência) (5,9,12). Os usuários foram classificados como tendo descontinuado o tratamento quando interromperam ou trocaram o medicamento biológico. 

A persistência – estar em tratamento – foi avaliada em 12 e 24 meses após a primeira dispensação. Na análise da persistência em 12 meses, foram incluídos os usuários que tiveram a oportunidade de serem acompanhados por esse período. Isso foi feito também para o cálculo da persistência em 24 meses. Essa medida permitiu analisar a proporção de participantes que persistiram no tratamento, em um e dois anos, e os fatores associados foram verificados. 

### Análise estatística

Estatísticas descritivas foram aplicadas para descrever as características demográficas e clínicas dos participantes. Proporções foram usadas para as variáveis categóricas como sexo, raça/cor de pele, índice de Gini, critérios modificados de Nova York, Assessment of SpondyloArthritis International Society e presença do HLA-B27. Para as variáveis contínuas, como idade, tempo de doença e tempo de uso do medicamento biológico, foram empregadas medidas de dispersão.

A ocorrência de dados faltantes em algumas variáveis foi inferior a 5,0%, portanto sem necessidade de imputação de dados, tendo em vista o reduzido impacto sobre as análises estatísticas. Para comparar as proporções de troca ou descontinuação entre os medicamentos, foi utilizado o teste qui-quadrado de Pearson. 

O modelo de riscos proporcionais de Cox foi utilizado para identificar fatores associados ao tempo até a descontinuação do primeiro medicamento biológico. O modelo considerou o *hazard ratio* ajustado, juntamente com o intervalo de confiança de 95%. As variáveis incluídas no modelo de Cox foram: idade, sexo, raça, indicador socioeconômico, status HLA-B27, duração da doença, sintomas clínicos e uso de medicamentos. A curva de sobrevida de Kaplan-Meier foi gerada para comparar o tempo até a descontinuação do tratamento, levando em conta a presença do HLA-B27. As diferenças entre os grupos foram analisadas por meio do teste de log-rank ao longo de todo o período avaliado. 

Para a avalição da existência de associação entre variáveis demográficas e clínicas com a persistência no tratamento, foram conduzidas análises univariadas utilizando o teste qui-quadrado de Pearson. A análise multivariada foi realizada por meio do modelo de regressão logística. Para a montagem do modelo, utilizaram-se as variáveis que apresentaram p-valor<0,200 no modelo univariado. Foi seguido o método de seleção de variáveis até a obtenção do modelo final. Por meio do teste de Hosmer-Lemeshow, foi avaliada a adequação do modelo. 

Foram utilizados o nível de significância (p-valor 0,050) para a análise multivariada e o teste qui-quadrado de Pearson. Todas as análises foram realizadas por meio do IBM SPSS Statistics, versão 26.

## Resultados

Entre 2018 e 2023, foram acompanhados e analisados 530 participantes. O tempo médio de acompanhamento foi 26 meses. 

Entre os participantes acompanhados, 265 (50,0%) eram do sexo masculino e 169 (31,9%) se autodeclararam brancos (Tabela 1). A idade média dos participantes foi 46,4 anos (desvio-padrão±13,17). O tempo médio de doença até a prescrição do primeiro medicamento biológico foi 5,9 anos (desvio-padrão±7,90). O Bath Ankylosing Spondylitis Activity Index médio dos participantes nesta coorte foi 6,9 pontos (desvio-padrão±1,52), o que indicou doença ativa. A alta atividade da doença também foi identificada por meio da ferramenta Ankylosing Spondylitis Disease Activity Score, com valores médios de 4,7 (desvio-padrão±2,1) (Tabela 1). 

**Tabela 1:** Características sociodemográficas e clínicas e perfil de utilização de medicamentos dos participantes com espondilite anquilosante em uso dos medicamentos biológicos. Minas Gerais, 2018-2023 (n=530)

Características	n (%)
**Sexo**	
Masculino	265 (50,0)
Feminino	265 (50,0)
**Raça/cor da pele**	
Branca	169 (31,9)
Pardo/preta	113 (21,3)
Outra/informação ignorada	248 (46,8)
**Faixa etária** (anos)	
18-44	235 (44,3)
45-59	206 (38,9)
≥60	89 (16,8)
**Índice de Gini**	
0,0000-0,4999	298 (56,2)
0,5000-1,000	232 (43,8)
**Tempo de doença** (anos) 0-5 ≥6	230 (43,4) 300 (56,6)
**Acometimento clínico**	
Entesite	430 (81,3)
Artrite periférica	114 (21,5)
Lombalgia inflamatória com início anterior aos 45 anos^a^	503 (94,9)
Sacroileíte em exames de imagem^a^	478 (90,2)
Presença do HLA-B27^a^	300 (56,6)
Lombalgia por mais de três meses^b^	315 (59,4)
Limitação dos movimentos da coluna lombar^b^	353 (66,6)
Expansão torácica diminuída^b^	166 (31,3)
Radiografia com detecção de sacroileíte bilateral de graus 3-4^b^	366 (69,1)
Radiografia com detecção de sacroileíte unilateral de graus 3 ou 4^b^	104 (19,6)
**Uso prévio de medicamentos para espondilite anquilosante**	
Anti-inflamatórios não esteroidais	525 (99,1)
Corticoides	137 (25,8)
Metotrexato	210 (39,6)
Sulfassalazina	202 (38,1)
Leflunomida	18 (3,4)
Hidroxicloroquina	13 (2,5)
**Primeiro medicamento biológico**	
Adalimumabe	198 (37,4)
Etanercepte	30 (5,7)
Infliximabe	80 (15,1)
Certolizumabe pegol	53 (10,0)
Golimumabe	159 (30,0)
Secuquinumabe	10 (1,9)

^a^Assessment of SpondyloArthritis International Society; ^b^Critérios modificados de Nova York.

Os medicamentos adalimumabe e golimumabe foram os mais prescritos, correspondendo a 37,4% e 30,0%. O etanercepte foi o inibidor fator de necrose tumoral menos prescrito, 5,7% (Tabela 1).

Os participantes em uso do infliximabe apresentaram maior proporção de descontinuação (27,5%). O teste qui-quadrado de Pearson demonstrou diferenças estatisticamente significativas para a interrupção do tratamento para os medicamentos infliximabe (maior taxa de interrupção) e golimumabe (menor taxa de interrupção) (p-valor<0,050). Para o desfecho troca, não foram encontradas diferenças estatisticamente significativas (Tabela 2).

**Tabela 2:** Número e proporção de troca e interrupção do primeiro medicamento biológico prescrito. Minas Gerais, 2018-2023 (n=530)

Medicamento	Prescrito	Troca	Interrupção
	n	n (%)	n (%)
Adalimumabe	198	43 (21,7)	34 (17,2)
Etanercepte	30	9 (30,0)	5 (16,7)
Infliximabe	80	10 (12,5)	22 (27,5)^a^
Certolizumabe pegol	53	12 (22,6)	8 (15,1)
Golimumabe	159	34 (21,4)	19 (11,9)^a^
Secuquinumabe	10	1 (10,0)	0 (0,0)
Total	530	109 (20,6)	88 (16,6)

^a^p-valor<0,050 no teste qui-quadrado de Pearson.

O tempo mediano até a descontinuação do tratamento foi 34,0 meses. No modelo de riscos proporcionais de Cox, o risco de descontinuação da terapia foi analisado como variável tempo-dependente. A análise revelou que a raça/cor de pele, a presença do HLA-B27 e a lombalgia inflamatória por mais de três meses apresentaram maior risco à descontinuação do tratamento.

A raça/cor da pele apresentou diferença estatisticamente significante (p-valor 0,045) com *hazard ratio* ajustado indicando que indivíduos com raça/cor da pele parda ou preta tinham risco 35,0% menor de descontinuar o tratamento em comparação aos de outras cores de pele. A presença do HLA-B27 também demonstrou associação significativa (p-valor 0,001), com risco 73,0% maior de descontinuação do tratamento entre indivíduos com HLA-B27 positivo (Figura 1). A lombalgia inflamatória por mais de três meses, embora com tendência à significância marginal (p-valor 0,053), aumentou em 40,0% o risco de descontinuação do tratamento (Tabela 3).

**Tabela 3:** *Hazard ratio* (HR) e intervalo de confiança de 95% (IC95%) ajustados pelas variáveis associadas à descontinuação da terapia: modelo de regressão de Cox. Minas Gerais, 2018-2023 (n=197)

Variáveis	Taxa de descontinuação n (%)	Mediana de tempo até a descontinuação (meses)	p-valor	HR ajustado (IC95%)^a^
Sexo			0,237	
Feminino	104 (39,2)	33,0 (31,15; 34,85)		
Masculino	93 (35,1)	34,0 (31,18; 36,82)		
**Faixa etária** (anos)			0,672	
18-44	85 (36,2)	33, 0 (30,87; 35,13)		
45-59	80 (38,8)	35,0 (30,90; 39,10)		
>60	32 (35,9)	33,0 (30,10; 5,90)		
**Raça/cor da pele**			0,045	
Branca	73 (43,2)	31,0 (25,15; 36,85)		1
Parda/preta	37 (32,7)	34,0 (29,61; 38,39)		0,65 (0,43; 0,96)
Outra/desconhecida	87 (35,1)	33,0 (30,93; 35,07)		0,76 (0,55; 1,04)
**Índice de Gini**			0,346	
0,0000-0,4999	108 (36,2)	35,0 (32,7; 37,33)		
0,5000-1,000	89 (38,4)	33,0 (29,95; 36,05)		
**HLA-B27**			<0,001	
Não	104 (45,2)	30,0 (25,92; 34,09)		1
Sim	93 (31,0)	35,0 (32,98; 37,02)		1,73 (1,30; 2,30)
**Tempo de doença** (anos)			0,896	
0-5	88 (38,3)	34,0 (30,7; 37,3)		
≥6	109 (36,3)	33,0 (30,7; 35,3)		
**Lombalgia inflamatória por mais de três meses**			0,053	
Não	88 (40,9)	33,0 (27,6; 38,4)		1
Sim	109 (34,6)	34,0 (32,2; 35,8)		1,40 (1,06; 1,86)
**Limitações dos movimentos da coluna lombar**			0,208	
Sim	126 (35,7)	33,0 (31,6; 34,4)		
Não	71 (40,1)	34,0 (24,2; 43,8)		
**Expansão torácica diminuída**			0,062	
Não	143 (39,3)	34,0 (31,5; 36,5)		1
Sim	54 (32,5)	33,0 (30,7; 35,3)		1,40 (1,03; 1,95)
**Sacroileíte unilateral**			0,610	
Sim	35 (33,7)	32,0 (29,3; 34,7)		
Não	162 (38,0)	35,0 (32,01; 37,9)		
**Sacroileíte bilateral**			0,273	
Sim	129 (35,2)	34,0 (32,4; 35,5)		
Não	68 (41,5)	33,0 (26,1; 39,8)		
**Uso prévio de corticosteroides**				
Sim	48 (35,0)	34,0 (32,5; 35,4)	0,121	
Não	149 (37,9)	33,0 (30,1; 35,9)		
**Primeiro medicamento biológico**			0,704	
Adalimumabe	77 (38,9)	33,0 (28,1; 37,9)		
Etanercepte	14 (46,7)	29,0 (17,5; 40,5)		
Infliximabe	32 (40,0)	35,0 (26,2; 43,8)		
Certolizumabe	20 (37,7)	33,0 (25,4; 40,6)		
Golimumabe	53 (33,3)	34,0 (32,3; 35,7)		
Secuquinumabe	1 (10,0)	-		

^a^Análise multivariada.

**Figura 1:** Kaplan-Meier com estimativa de descontinuação terapêutica associada à presença do HLA-B27 em 60 meses. Minas Gerais, 2018-2023 (n=530)

Ao analisar a relação das variáveis demográficas e clínicas da linha de base com a persistência em 12 e 24 meses de acompanhamento, foram observadas diferenças estatisticamente significativas em participantes com HLA-B27, lombalgia inflamatória por mais de três meses e expansão torácica diminuída (p-valor≤0,050) (Tabela 4). Os resultados aos 12 meses revelaram que pacientes com expansão torácica diminuída apresentaram redução de 55,0% nas chances de persistência terapêutica. O HLA-B27 foi identificado como fator de risco, apresentando redução de 60,0% nas chances de persistência terapêutica em comparação àqueles com teste negativo. A presença de lombalgia inflamatória por mais de três meses mostrou-se associada à diminuição de 48,0% nas chances de persistência terapêutica. Em 24 meses de acompanhamento, apenas o sintoma clínico de expansão torácica demonstrou associação significativa com a persistência ao tratamento (p-valor 0,052) (Tabela 4).

**Tabela 4:** Razão de chances (*odds ratio*, OR) e intervalo de confiança de 95% (IC95%) ajustados pelas variáveis associadas à persistência em 12 e 24 meses. Minas Gerais, 2018-2023 (n=530)

Variáveis	Persistência 12 meses (n=530) %	p-valor	OR ajustado (IC95%)^a^	Persistência 24 meses (n=332) %	p-valor	OR ajustado (IC95%)^a^
Geral	82,1			63,0		
Sexo		0,910			0,610	
Feminino	81,9			61,6		
Masculino	82,3			64,3		
**Faixa etária** (anos)		0,418			0,997	
18-44	83,8			63,0		
45-59	82,0			63,1		
>60	77,5			62,5		
**Raça/cor da pele**		0,664			0,393	
Branca	81,1			58,3		
Parda/preta	85,0			62,3		
Outra/desconhecida	81,5			66,7		
**Índice de Gini**		0,747			0,237	
0,0000-0,4999	82,6			65,5		
0,5000-1,000	81,5			59,1		
HLA-B27		0,001			0,066	
Sim	87,0		1	67,2		1
Não	75,7		0,40 (0,25; 0,64)	57,3		0,63 (0,40; 0,99)
**Tempo de doença** (anos)		0,334			0,330	
0-5	80,7			60,7		
≥6	83,9			66,0		
**Lombalgia inflamatória por mais de três meses**		0,016			0,141	
Sim	85,4		1	66,2		
Não	77,2		0,52 (0,33; 0,82)	58,2		
**Limitações dos movimentos da coluna lombar**		0,132			0,414	
Sim	83,9			64,4		
Não	78,5			59,8		
**Expansão torácica diminuída**		0,017			0,052	
Sim	88,0		1	70,4		1
Não	79,4		0,45 (0,26; 0,79)	59,4		0,59 (0,36; 0,96)
**Sacroileíte unilateral**		0,108			0,510	
Sim	87,5			66,7		
Não	80,8			62,1		
**Sacroileíte bilateral**		0,377			0,168	
Sim	83,1			65,4		
Não	79,9			57,4		
**Uso prévio de corticosteroides**		0,508			0,044	
Sim	83,9			70,8		
Não	81,4			59,3		
**Primeiro medicamento biológico**		0,412				
Adalimumabe	82,3					
Etanercepte	73,3					
Infliximabe	85,0					
Certolizumabe	77,4					
Golimumabe	82,4					
Secuquinumabe	100,0					

^a^Análise multivariada.

## Discussão

Foi avaliado o desempenho dos medicamentos biológicos utilizados no tratamento da espondilite anquilosante no SUS, em Minas Gerais, com base nos dados obtidos dos processos administrativos do componente especializado da assistência farmacêutica. 

Os participantes que compuseram esta coorte possuíam idade média e distribuição de gênero semelhantes às observadas anteriormente no Brasil (13,14). O tempo médio de doença foi próximo ao observado em outras coortes brasileiras, nas quais o tempo médio da doença foi 7,6 anos (13,14). Os resultados confirmaram que o início da espondilite ocorre, em média, na terceira década de vida, com agravamento clínico se manifestando cerca de seis anos após o diagnóstico.

A maioria dos participantes se autodeclarou branca, e parte não quis declarar. Entre 53,0% e 56,0% dos usuários com espondilite anquilosante, não se autodeclaram brancos (14,15). Globalmente, os dados epidemiológicos disponíveis sugerem maior prevalência da doença em pessoas brancas. Entretanto, esses estudos foram majoritariamente realizados em países europeus e norte-americanos, com populações pouco comparáveis à população brasileira (16,17). A lacuna de dados sobre raça/cor da pele no contexto brasileiro limitou a compreensão do impacto da doença nesse grupo populacional.

O índice de Gini foi utilizado para representar a condição socioeconômica dos participantes com base na desigualdade de renda em seu município de residência. Sua interpretação pode ser somada ao índice de desenvolvimento humano, que mensura o desenvolvimento municipal por meio de indicadores de saúde, educação e renda per capita. Em Minas Gerais, o índice de desenvolvimento humano em 2021 foi 0,774, o que posicionou o estado entre os quatro com maior índice do país (18). Esse resultado esteve alinhado à distribuição média alcançada pelo índice de Gini neste estudo.

Pouco mais da metade dos participantes apresentaram o HLA-B27. Esse número divergiu dos achados descritos na literatura internacional. Uma revisão sistemática sumarizou os resultados norte-americanos e de países europeus (19). Neste estudo 70,0% e 94,0% dos participantes tinham o HLA-B27 (19). Na Espanha, também foi observado que 75,0% dos participantes de determinada coorte possuíam o HLA-B27 (20).

Neste estudo, a baixa prevalência do HLA-B27 pode estar associada a fatores genéticos e étnicos específicos da população avaliada.

Outras análises envolvendo o antígeno foram conduzidas. Entre estas, destaca-se o HLA-B27 como fator associado ao maior tempo até a descontinuação e à maior persistência no tratamento. Discute-se o papel do HLA-B27 na patogênese da doença, no entanto a relação entre a presença do HLA-B27 e o pior prognóstico da doença é pouco abordada. A presença do alelo HLA-B27 demonstrou pior prognóstico e início mais precoce dos sintomas da doença, conforme demonstrado em estudos com amostras de 150 e 1.235 participantes (21,22).

O adalimumabe foi o mais prescrito para ser utilizado como primeira escolha (13,14,23). A manutenção do adalimumabe como primeira opção pôde indicar a preferência dos prescritores por este tratamento e os benefícios alcançados com o seu uso tanto no cenário nacional quanto internacional (23).

Os casos de prescrição do secuquinumabe como primeira escolha vão em desencontro com os critérios estabelecidos no Protocolo Clínico e Diretrizes Terapêuticas (5). As justificativas para uso do medicamento incluíram resultado positivo para prova tuberculínica, manifestações extra-articulares, falha ao uso de anti-inflamatórios não esteroidais e medicamentos modificadores do curso de doença sintéticos. O protocolo recomenda o uso do secuquinumabe seja exclusivamente após a falha terapêutica com um inibidor fator de necrose tumoral (5). Relataram-se o uso do secuquinumabe na primeira linha de tratamento e os benefícios relacionados ao uso do medicamento, entre eles a maior persistência e a melhora da atividade da doença (24). 

As recomendações internacionais Assessment of SpondyloArthritis International Society e European League Against Rheumatism de 2016 aprovaram o uso do secuquinumabe apenas para pessoas com espondiloartrite axial e com sacroileíte radiográfica. Na atualização de 2022, o critério foi alterado, e o secuquinumabe foi indicado como primeira linha de tratamento para espondiloartrite axial (25). Essa atualização reforçou que o medicamento vem se destacando no tratamento da doença, demonstrando ser eficaz e seguro tanto quanto os inibidores fator de necrose tumoral.

A troca de medicamento ocorreu com maior frequência entre os participantes em uso do etanercepte. Neste estudo, a taxa de descontinuação observada foi inferior à relatada anteriormente (13,26). Identificou-se que a principal causa de descontinuação (50,0%) estava relacionada à ineficiência terapêutica, enquanto 30,0% foram atribuídas reações adversas a medicamentos (26). 

Nesta coorte, pessoas com HLA-B27 ou lombalgia inflamatória por mais de três meses apresentaram maior risco para a descontinuação do tratamento. Os resultados sugeriram a associação entre a gravidade da doença e o pior desempenho de alguns medicamentos, destacando a importância de se considerar fatores clínicos no manejo da persistência ao tratamento. A descontinuação ou interrupção da terapia pode resultar no aumento de gastos públicos, dado o possível agravamento destes usuários. Estratégias como a educação dos usuários sobre os riscos da interrupção terapêutica podem ser úteis. Para casos mais graves, nos quais a doença apresenta pior prognóstico, é essencial reforçar a necessidade de persistência ao tratamento, visando estabilizar a condição e evitar complicações a longo prazo. 

A persistência ao tratamento pode ser considerada indicador de efetividade, tolerância e adesão à terapia medicamentosa (8,27). Em uma coorte com 19.319 usuários brasileiros e canadenses, identificou-se que o tempo médio de persistência dos participantes em uso de adalimumabe, etanercepte e infliximabe foi dez meses (14). Evidenciou-se que 52,0% a 65,0% dos participantes em uso de infliximabe, etanercepte e adalimumabe persistiram até o primeiro ano (14). Em outra coorte brasileira, com 1.251 usuários, 80,0% dos indivíduos persistiram no primeiro ano de acompanhamento, enquanto 60,0% estavam em uso do biológico ao final do segundo ano (13). Apesar das diferenças encontradas entre os estudos, a falta de persistência após o primeiro ano de tratamento levanta questionamentos sobre a possível falta de efetividade terapêutica desses medicamentos após um ano de uso.

Este estudo apresentou limitações inerentes às pesquisas de mundo real, especialmente aquelas que utilizam dados secundários. Os dados analisados foram obtidos de processos administrativos de solicitação de medicamentos de alto custo. Esses documentos são preenchidos por médicos especialistas e suas informações são atestadas por meio dos resultados de exames de imagem e laboratoriais anexados ao processo. De acordo com as exigências do protocolo, o histórico de uso prévio de medicamentos biológicos deve ser registrado no laudo de solicitação. Possíveis inconsistências ou omissões no preenchimento podem limitar a completude e a precisão dos dados. 

Medidas para reduzir o risco de viés de informação foram aplicadas, mas é importante ressaltar que a ausência de dados clínicos, como comorbidades e uso de outros medicamentos, limitam a análise detalhada de fatores clínicos, que pode impactar diretamente na persistência ao tratamento. Além da carga de comorbidades, fatores comportamentais também podem influenciar a persistência ao tratamento. Aspectos como adesão ao tratamento, acesso regular aos serviços de saúde e hábitos de vida podem impactar os desfechos terapêuticos, mas não são sistematicamente documentados nos registros administrativos utilizados. Essas limitações devem ser consideradas na interpretação dos achados.

Outra limitação está associada à forma como o status socioeconômico foi avaliado, utilizando exclusivamente o índice de Gini. Embora esse indicador meça a desigualdade de renda dentro de uma cidade, ele não reflete diretamente a renda absoluta, o acesso aos serviços de saúde ou a qualidade de vida individual dos usuários. A impossibilidade de coletar outros indicadores, como renda per capita, acesso a serviços de saúde ou nível educacional, é decorrente da limitação dos dados disponíveis nos formulários analisados. O índice de Gini não permite inferir, por exemplo, se usuários com maior poder aquisitivo e melhor acesso à saúde são os principais usuários dessas terapias.

Minas Gerais, devido à sua extensão territorial e população diversificada, pode refletir a realidade nacional. Coletados rotineiramente, os dados administrativos do SUS são fonte valiosa para estudos do mundo real, permitindo a visão detalhada da epidemiologia e clínica dos usuários. Sua análise fornece informações essenciais sobre a efetividade e segurança dos tratamentos, o que auxilia na tomada de decisões em saúde pública. A ampla disponibilidade desses dados viabiliza estudos populacionais mais representativos, possibilitando a identificação de desfechos de saúde e fatores de risco com maior precisão.

Este estudo destacou a utilidade dos dados administrativos da assistência farmacêutica como ferramenta para monitorar o perfil clínico e a persistência terapêutica de usuários com espondilite anquilosante no SUS. Fatores clínicos, como HLA-B27 e sintomas da doença, apresentaram maior risco de descontinuação do tratamento com medicamentos biológicos. Entretanto, em 24 meses de acompanhamento, foi observada associação de persistência associada ao HLA-B27 positivo. Isso pode indicar que a persistência ao tratamento pode ser tempo dependente. Por outro lado, aspectos demográficos, como a raça/cor de pele, demonstraram efeitos protetores. Os padrões observados de prescrição e troca de medicamentos biológicos apontaram para variações nas taxas de persistência e descontinuação, que podem refletir diferenças tanto no manejo clínico quanto nas características dos usuários. Esses achados reforçam a necessidade de integrar fatores clínicos e sociodemográficos na gestão personalizada da espondilite anquilosante, contribuindo para melhores desfechos terapêuticos. 

## Data Availability

O banco de dados e os códigos de análise utilizados na pesquisa estão disponíveis mediante solicitação aos autores.
